# MOF-Derived FeS/C Nanosheets for High Performance Lithium Ion Batteries

**DOI:** 10.3390/nano9040492

**Published:** 2019-03-30

**Authors:** Jianguo Zhao, Zhuan Hu, Dezhu Sun, Hong Jia, Xianming Liu

**Affiliations:** 1 School of Physics and Electronic Information, Luoyang Normal University, Luoyang 471934, China; huzhuan1119@163.com (Z.H.); sundezhu1997@163.com (D.S.); 2 Henan Key Laboratory of Electromagnetic Transformation and Detection, Luoyang Normal University, Luoyang 471934, China; 3 School of Chemistry and Chemical Engineering, Luoyang Normal University, Luoyang 471934, China; lxm-nanoenergy@lynu.edu.cn

**Keywords:** FeS/C, cycle stability, rate performance, synergistic effects

## Abstract

In recent years, transitional metal sulfides have received much attention as lithium ion batteries (LIBs) anode. In this paper, FeS/C nanosheets are prepared through Fe-based metal organic frameworks (Fe-MOFs) as a precursor. The electrochemical performance of FeS/C nanosheets has been obviously improved due to the synergistic effect of the oxygen doped carbon and the special flake morphology. When the test current density is 0.1 A/g, the initial discharge capacities of FeS/C nanosheets is up to 1702 mAh/g and can retain reversible capacities of about 830 mAh/g over 150 cycles with the voltage ranging from 0.01 V to 3 V. Moreover, these composite materials are proved to have a good rate performance and the capacities reach 460 mAh/g even at a higher current density of 5 A/g. This work suggests that FeS/C nanosheets are excellent anode materials for LIBs.

## 1. Introduction

Nowadays, the world faces more and more serious energy and environmental problems. The conventional fossil energy gradually dries up, and it puts enormous pressure on the environment in its utilizing process. People are starting to exploit renewable solar, wind, oceanic energy, and so on [1,2,3]. However, the most critical issue is the large-scale energy storage systems to make utilization of renewable energy. Among them, Rechargeable LIBs are thought to be the best energy storage devices because of their high energy density, high power density, safety, portability, and excellent cycling stability against other storage devices [4,5,6,7,8]. However, the specific capacity is 372 mAh/g corresponding to the commercial graphite as the anode of LIBs [9,10]. With the development of different kinds of energy consumption devices, the commercial graphite cannot meet the demand. Therefore, a lot of efforts have been performed to look for electrode materials with higher specific capacity, power density, Coulombic efficiency, better conductivity, and cycling stability to replace the commercial graphite [11,12,13,14,15,16].

In all kinds of electrode materials, metal sulphides are widely studied due to their high capacity as the anode of LIBs [17,18,19,20,21,22]. Iron monosulfide (FeS) has drawn extensive attention as promising electrode materials because of the characteristics of high energy density, low-budget, safety, and richness of raw materials [23,24]. The critical problems for FeS as electrode materials are the poor cycle performance, large volume change, and the appearance of soluble polysulfide causing the lost of active materials. To address these issues, a lot of groups proposed different ways to solve these problems through introducing of conductive layers or reducing the size of FeS. S. S. Zhang et al. [25] pointed out that carbon precursor coated iron monosulphide microcrystals possessed outstanding rate performance due to the small particle (<1 µm). Yu et al. [26] prepared FeS@C/carbon cloth by hydrothermal method and their sample shows elevated cyclic stability and rate performance owing to the special structure and conductive carbon cloth. Tour et al. [27] fabricated FeS nanoparticles coated by graphene with high electrochemical performances owing to the synergy between the external graphene and internal FeS. However, the effect of doping ionics on the coated carbon for the performance of LIBs has scarcely been reported.

Metal organic frameworks (MOFs) are a kind of crystalline sponge-like materials formed by i inorganic nodes and multitopic organic linkers [28]. Up to now, MOFs have been widely explored in various applications including catalysis, drug delivery, optoelectronics, and electrochemical fields, etc. [29,30,31,32,33,34] due to the superiority of controllable structures, huge surface area, tunable pore size, and high porosity. It is generally accepted that MOFs are not suitable for the electrochemical application as electrodes because of their poor electronic conductivity. Moreover, MOFs has been used as an important alternative precursor to prepared carbon materials, metal oxides, metal sulfides, metal phosphides, and metal selenides as the anode of LIBs, owing to the advantages mention above, which are beneficial to enhance the electrochemical reaction kinetics, dominate the buffer of volume changes, and further improve the electrochemical performance [35,36,37,38,39]. Among all types of MOFs, Fe-MOFs are ideal candidates for the precursor materials in LIBs because iron is cheap, widely distributed, and is a less toxic element.

In this paper, Fe-MOFs derived FeS/C nanosheets were designed and fabricated as the anode of LIBs. It shows significantly improved cyclic capacity, cyclic stability, and excellent rate capacity. The specific capacity of FeS/C nanosheets maintains 830 mAh/g after 150 cycles at a current density of 0.1 A/g. Even on the high current density of 5 A/g, it still delivers a specific capacity of 460 mAh/g.

## 2. Experimental

Preparation of the Fe-MOFs: The detailed preparation process of Fe-MOFs has been reported by other researchers [40]. The chemicals in the work were of analytical grade without further purification and are purchased by Sinopharm Chemical Reagent Co., Ltd. (Shanghai, China). Firstly, 1.08 g of FeCl_3_·6H_2_O and 0.88 g of 1,4-dicarboxybenzene were added into 54 mL N,N-dimethylformamide (DMF) and stirred to form yellow solution. Secondly, 6 mL of 0.4 M NaOH solution was put into the obtained yellow solution and stirred for another 15 min. The whole mixture solution was transferred to a 100 mL Teflon-lined stainless steel autoclave and heated in an oven at 100 °C for 24 h. After that, the mixture solution cooled to room temperature naturally. Finally, the samples were cleaned with ethanol several times and was in vacuum freeze-drying for 12 h.

Preparation of the FeS/C nanosheets: The two raw materials of Fe-MOFs and sublimed sulfur (the weight ratio of Fe-MOF and sublimed sulfur is 1:1.5) were mixed and grinded for 15 min, then transferred to a quartz tube. The mixture was directly calcinated at 450 °C with a heating rate of 2°/min in a tubular furnace under argon for 4 h. At last, the black power of FeS/C nonosheets were obtained.

**Characterization.** The crystal structure and morphologies of as-obtained samples was investigated by X-ray diffraction (XRD, Bruker D8 Advance, Bill ricard, MA, USA) with a Cu Kα radiation, Raman scattering spectra (JY-HR800, Jobin Yvon Company, Lille, France) using an Ar^+^ laser (514.5 nm) as the excitation line, scanning electron microscopy (SEM, Carl Zessi, Sigma HD, Jena, Germany) equipped with energy-dispersive X-ray spectrometer (EDS), and transmission electron microscopy (TEM, JEOL JEM-2100F, Tokyo, Japan). The specific surface of FeS/C nanosheets was characterized by Brunauer-Emmett-Teller (BET, ASAP 2020, Micromeritics, Norcross, GA, USA) N_2_ absorption- desorption.

**Electrochemical Measurements.** 80% of active material (FeS), 10% of acetylene black, and 10% of polyvinylidene fluoride (PVDF) by weight were mixed in N-methyl-2-pyrrolidone (NMP) to form a homogeneous slurry. The slurry was then pasted on the copper foil, and dried under vacuum at 60 °C overnight. The CR2032 coin cells were assembled in a glove box filled with dry argon gas. The electrolyte was used as 1 M LiPF_6_ in 1:1 ethylene carbonate (EC) /dimethyl carbonate (DMC). The cyclic voltammetry (CV) and electrochemical impedance spectroscopy (EIS) of the coin cells were performed on an electrochemical workstation (CHI660E). The galvanostatic charge and discharge cycling measurement were carried out on a battery-testing system (Shenwei-CT2001A). The measurement was evaluated at an open circuit potential with a modulation amplitude of 5 mV and a frequency range from 0.01 Hz to 100 kHz.

## 3. Results and Discussion

**Scheme 1:**
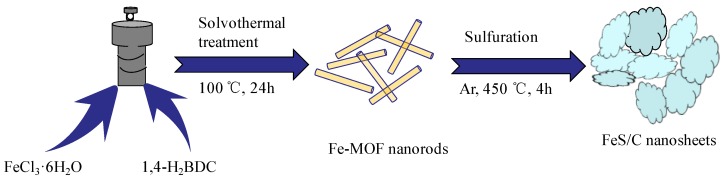
Schematic illustration of the formation process of FeS/C nanosheets.

Scheme 1 shows the detailed preparation process of Fe-MOFs derived FeS/C nanosheets. First, needle like Fe-MOF nanorods were obtained by solvothermal process. The crystalline structure of Fe-MOFs is characterized by the X-ray diffraction analysis (XRD) (see Figure S3, Supplementary Materials). The XRD patterns of the as-prepared product confirm that a crystallization pure phase of MIL-100(Fe) has been obtained as the diffraction peaks is consistent with the other reported [40,41]. The FT-IR spectrum further supports the results of XRD. The strong and broad peak located at 3400 cm^−1^ is assigned to the O-H vibration. The characteristic absorption peaks of the Fe-MOF were observed at 1672, 1596, 1394, 1053, and 747 cm^−1^, which could mainly originate from the vibrations of carboxylate groups and are close to those reported in the previous literatures. In addition, the peak at 552 cm^−1^ is related to the Fe-O stretching vibration. Compared to the reference, the peak has blue-shift because our precursor is 1,4-dicarboxybenzene. The results show that MOF structure is formed by the Fe ions and 1,4-dicarboxybenzene organic ligands. The template of Fe-MOFs is composed of Fe^3+^ ions and the organic ligands via strong chemical bonds with high surface area. Then, the mixture of these obtained nanorod-like Fe-MOFs and sublimed sulfur were calcined in a tubular furnace in argon atmosphere and the solid nanorods transformed into nanosheets. In the process, the sulfur was sublimed into gaseous sulfur, and reacted with the Fe−O clusters in Fe-MOF to form the nanoparticles of FeS. Meanwhile, the carbon linkers in Fe-MOF were cracked into oxygen-doped carbon that coated on the FeS. A similar result has been reported in the other paper [42] and consists of the results of XRD. It can form good crystalline FeS coated by oxygen-doped carbon combined with sublimed sulfur through the strong metallic bonding.

**Figure 1:**
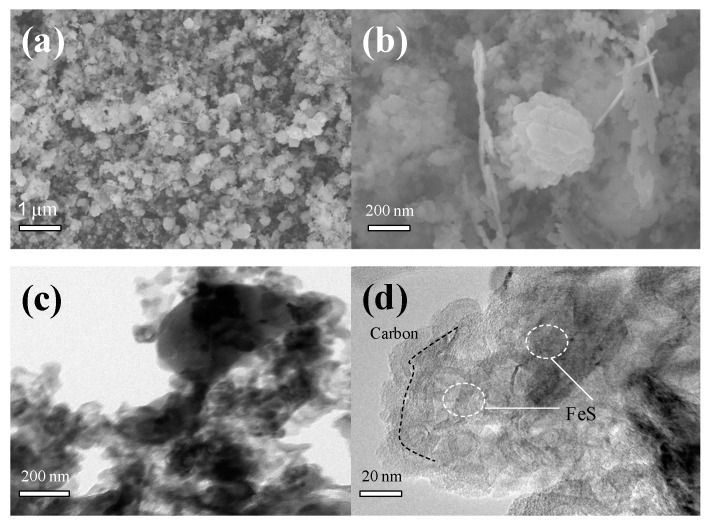
SEM images of (**a**) low- and (**b**) high-magnification of FeS/C nanosheets transformed from Fe-MOFs nanorods and TEM images of (**c**) low and (**d**) high-magnification of the FeS/C nanosheets transformed from Fe-MOFs nanorods.

Figure 1 and Figure S1 show the SEM images of of FeS/C and Fe-MOF. The Fe-MOFs show a regular cone capped nanorods with a high crystal quality and their length and diameter are about 750 nm and 150 nm, respectively (Figure S1b,c). There are still some nanoparticles because the whole response system is in a state of unsteady diffusion resulting in the insufficient or inhomogeneous reaction. After heat treatment, Fe-MOF nanorods convert to flake morphology due to the formation of FeS nanoparticles and coating carbon, as shown in Figure 1a,b. Besides, the nanosheets are extremely thin that can be beneficial to make full use of the active materials. We further performed the EDS mapping of FeS/C nanosheets shown in Figure S2a–e. The element distribution of carbon, iron, oxygen, and sulfur is featured as expected. The microstructure of FeS/C nanosheets is further investigated by TEM and HRTEM. The TEM image (Figure 1c) of FeS/C nanosheets displays the well-defined FeS/C flakes. It is consistent with the results obtained of SEM images. The characteristics of carbon-encapsulated FeS nanoparticles were confirmed by HRTEM images. FeS particles were surrounded by carbon, as shown in Figure 1d. The forming reason of carbon-coated FeS is due to the in-situ decomposition of organic frameworks. Additionally, these fully encapsulated FeS nanoparticles have fine crystallinity corresponding to the external amorphous carbon. This unique core-shell structure of FeS/C nanosheets may availably improve electron conductivity and specific capacity.

**Figure 2:**
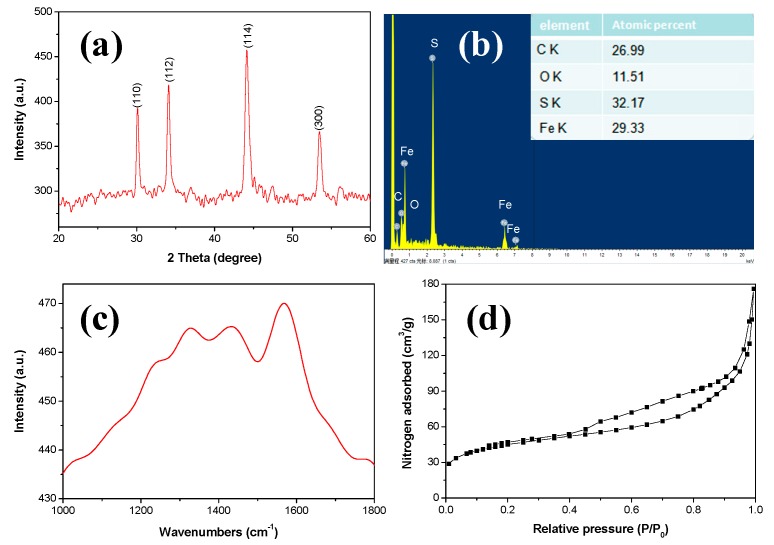
(**a**) XRD pattern, (**b**) EDS pattern, (**c**) Raman spectrum, and (**d**) BET spectrum of FeS/C nanosheets transformed from Fe-MOFs nanorods.

The XRD pattern of FeS/C nanosheets is shown in Figure 2a. All the diffraction peaks at 30.2°, 34.1°, 44.1°, and 53.5° are observed, which are assigned to (110), (112), (114), and (300), respectively. This result is in accordance with the hexagonal iron sulfide (ICDD 37-0477). The strong and obvious diffraction peaks indicate that the FeS/C nanosheets possess polycrystalline structure. The grain size calculates from the XRD pattern using Scherrer equation is about 25 nm. In order to further evaluate the ratio of Fe and S elements, the EDS pattern of FeS/C nanosheets is measured and shown in Figure 2b. From the experimental pattern, we can see that the atomic ratio of iron/sulfur is about 1:1. In order to further verify the existence of surface carbon, we analyze the structure of FeS/C using Raman spectroscopy (Figure 2c). There are two peaks at around 1327 cm^−1^ (D band) and 1569 cm^−1^ (G band) in the Raman spectrum of FeS/C, indicating that carbon in the composite is amorphous [43]. The other two peaks located at about 1230 and 1450 cm^−1^, were reported by other researchers of the vibrations of sp^2^ C-H chains and to the C–C stretching vibrations (G band) of sp^2^ clusters with large π electron delocalization length [44].

To detect the pore geometry of FeS/C nanosheets, nitrogen adsorption-desorption studies are performed. Figure 2d shows the typical N_2_ adsorption−desorption isotherm. From this figure, it can be seen that in the very low range of P/P_0_, the N_2_ adsorption quantity has a sharp increase because of the single layer adsorption in the micropores. In other P/P_0_ ranges, the type of adsorption is the same as H3-type hysteresis loop, indicating the multilayer adsorption owing to the mesopores and macropores [45]. Furthermore, the specific surface area of FeS/C is about 150 cm^2^/g.

**Figure 3:**
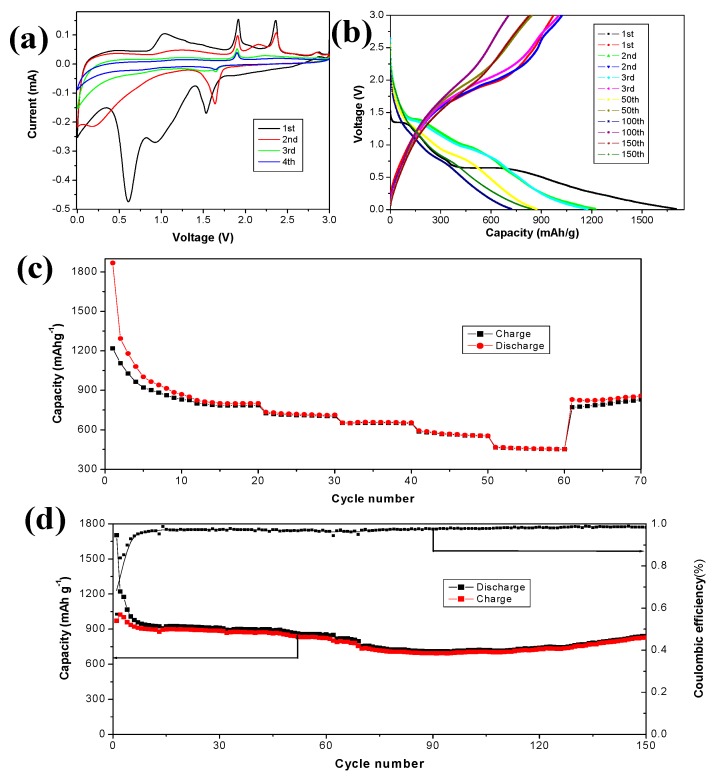
(**a**) Cyclic voltammetry curves of FeS/C nanosheets with a cutoff voltage ranging from 0.01–3.0 V (vs. Li/Li^+^) at 0.1 mV/s. (**b**) The galvanostatic charge/discharge profiles of FeS/C nanosheets electrode with a cut-off voltage ranging from 0.01–3.0 V (vs. Li/Li^+^) at the current density of 0.1A g^−1^. (**c**) Rate performance of FeS/C nanosheets transformed from Fe-MOFs nanorods. (**d**) Cycle performance of FeS/C nanosheets transformed from Fe-MOFs nanorods at 0.1 A g^−1^.

The half-cells are assembled with FeS/C as the anode electrodes to illustrate the LIBs capacity of FeS/C nanosheets. Figure 3a shows the CV curves of FeS/C nanosheets at a scanning rate of 0.1 mV s^−1^ with the range from 0.01 to 3 V of FeS/C nanosheets for the first four cycles. For the first discharge cycle, the reduction peak is located at 0.6 V, which can be ascribed to the formation of solid electrolyte interface (SEI) layer due to the decomposition of electrolyte. The plateau at 0.9 V corresponds to the formation of Li_2_S and Fe (Li_2_FeS_2_ + 2Li^+^ + 2e^−^ = 2Li_2_S + Fe) and the third reduction peak is at about 1.5 V owing to the reduction of Li to form Li_2_FeS_2_ (2FeS + 2Li^+^ + 2e^−^ = Li_2_FeS_2_ + Fe). For the first charge cycle, there are two oxidation peaks at 1.9 V and 2.3 V due to the oxidation process from Fe to FeS (Fe + Li_2_S − 2e^−^→2Li^+^ + FeS) and the formation of Li_2_FeS_2_ (Fe + Li_2_S − 2e^−^→2Li^+^ + Li_2_FeS_2_), respectively. After the first cycle, the peak from the SEI film is irreversible, indicating that the carbon on the surface makes the SEI layer more stable and the capacity loss smaller due to the formation of the SEI layer. From the second to fourth scans, the cathodic peaks are shifted to 1.63 V and the curves are almost identical indicating that there is a good reversibility. Similar results have also been reported in other article using FeS/C composite material as the anode of LIBs^22^.

The discharge-charge curves of FeS/C with the voltage ranging from 0.01 V to 3 V at a current density of 0.1 A/g for the cycles between the 1st and 150th are shown in Figure 3b. For the first discharge curve, there are three plateaus located at 0.6 V, 0.9 V and 1.5 V, respectively. These results are in accord with the first reduction curve of the CV. Additionally, the initial discharge capacity of FeS/C nanosheets is about 1702 mAh/g. The loss of capacity for FeS/C nanosheets at the initial discharge curve may be due to the decomposition of the electrolyte, non-reversible reaction of electrode materials, and the SEI layer formation [46,47].

Figure 3c shows the rate performance of FeS/C nanosheets for the current density from 0.1 A/g to 5 A/g. It is obvious that FeS/C nanosheets have outstanding rate capability. The discharge specific capacities of FeS/C nanosheets are about 930, 799, 715, 662, 566, and 460 mAh/g at the current density of 0.1, 0.2, 0.5, 1.0, 2.0, and 5.0 A/g, respectively. The specific capability can still deliver about 830 mAh/g, when the current density returns to 0.1 A/g.

Figure 3d shows the electrochemical cyclability of FeS/C corresponding to 150 cycles at 0.1 A/g with the potential ranging from 0.01 to 3 V. For the initial cycle, the discharging capacity is up to 1702 mAh/g and the charging capacity is 972 mAh/g. It shows the first Coulombic efficiency about 57% due to the inevitable decomposition of electrolyte and formation of SEI film. Then, the capacity gradually decreases to 694 mAh/g during the initial dozens of cycles. At last, the capacity continues to increase and keeps a stable value of 830 mAh/g corresponding to the coulombic efficiency of over 98.0% after 150 cycles. This variation of capacity is attributed to the activation process of FeS/C as the anode material at the initial dozens of cycles. At the beginning of first few cycles, the specific capacity demonstrates an irreversible loss because of the side effect of Li inserted into FeS/C electrode and the formation of an SEI film during the degradation of electrolyte. As the SEI film becomes stable, the electrode will be reactivated. The specific capacity of FeS/C is tending to stabilize.

**Figure 4:**
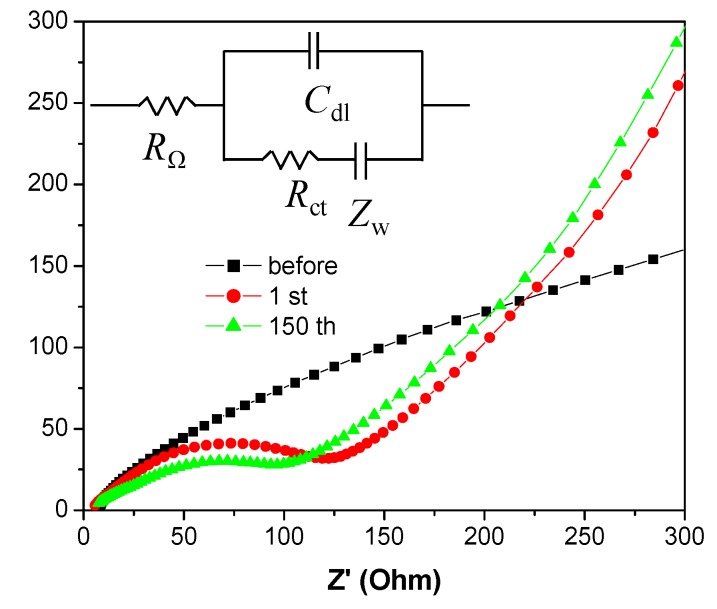
EIS spectra of FeS/C nanosheets electrodes after different cycles (inset: selected equivalent circuit) obtained with an amplitude of 5 mV in a frequency range from 0.01 Hz to 100 kHz.

To further illustrate the superior electrochemical performance of our FeS/C nanosheets, the electrochemical impedance spectrum (EIS) of this composite materials are measured before and after cycling, as shown in Figure 4. The equivalent circuit model of the EIS is shown in the inset of Figure 4. *Rs* represents the physical resistance of the coin cell, *C_dl_* signifies the constant phase element due to capacitance dispersion and *R_ct_* denotes charge-transfer resistance. While *Z_w_* gives Warburg resistance corresponding to the Li^+^-ion diffusion in the composite material. A semicircle appears after cycles at the high frequency corresponds to the R_ct_ and a sloping line at the low frequency corresponds to *Z_w_* due to the Li^+^-ion diffusion, respectively [48]. The *R_ct_* values of 179, 118, and 106 Ohm correspond to the values of before cycle, cycle 1st, and cycle 150th, respectively. Apparently, the *R_ct_* of FeS/C nanosheets after cycling is much smaller than before cycling. This indicates that the electron transfer rate has significantly improved after cycling.

The excellent electrochemical properties may be attributed to the following advantages of the active material according to the above analysis. Firstly, the content of oxygen atoms might induce numerous defects and change the growth dynamics of the coating carbon, resulting in a bigger interlayer distance. Evidently, the enlarged carbon interlayer distance of the sample was good news for the fast Li-ion insertion/extraction and enhanced electrochemical performances. Meanwhile, the oxygen heteroatoms provide additional reaction sites and the electrochemical performance can also be improved [49]. Secondly, the FeS/C nanosheets have a high surface area that can enhance the contacts between the electrolyte and the electrode materials. It accelerates the transfer of electrolyte through the FeS/C materials and improves the rate properties and cyclability [50]. Thirdly, the existence of amorphous carbon in FeS/C nanosheets can improve the conductivity of the iron electrode. Additionally, elemental sulfur can sometimes promote the dissolution of iron active materials and prevent the rapid electrode passivation, resulting in improved cycle performance [51].

## 4. Conclusions

In summary, the electrochemical properties of FeS/C nanosheets derived from Fe-MOFs are systematically investigated as anode of LIBs using CV, galvanostatic charging-discharging cycles, and EIS. The composite materials deliver a large discharge capacity of 1702 mAh/g in the initial cycle and retain 830 mAh/g over 150 cycles at 0.1 A/g. Meanwhile, our FeS/C materials also have an excellent rate performance. The outstanding electrochemical performance may be owing to the combined effect of oxygen doped carbon and nanosheet morphology. FeS/C is therefore a good anode for LIBs.

## Supplementary Materials

The following are available online at https://www.mdpi.com/2079-4991/9/4/492/s1, Figure S1: SEM images of (a) low- and (b) high-magnification of the Fe-MOFs nanorods. Figure S2: The elemental mapping images of FeS/C nanosheets. Figure S3: XRD pattern of uniform Fe-MOF nanorods, Figure S4: FTIR pattern of uniform Fe-MOF nanorods. Table S1: Recent reports on the electrochemical data of FeS as anode material for lithium-ion batteries.
