# Supplementary material for: MOF-Derived FeS/C Nanosheets for High Performance Lithium Ion Batteries
Source: Nanomaterials (Basel). 2019 Mar 30;9(4):492. doi: 10.3390/nano9040492 (PMC6523760; doi:10.3390/nano9040492)
Supplement: Supplementary file 1 [file nanomaterials-09-00492-s001.pdf]

MOF-Derived FeS/C Nanosheets for High Performance Lithium Ion Batteries

Jianguo Zhao [1,2,*], Zhuan Hu [1,2], Dezhu Sun [1,2], Hong Jia [1,2,*] and Xianming Liu [3]

[1] School of Physics and Electronic Information, Luoyang Normal University, Luoyang 471934, China; huzhuan1119@163.com (Z.H.); sundezhu1997@163.com (D.S.)
[2] Henan Key Laboratory of Electromagnetic Transformation and Detection, Luoyang Normal University, Luoyang 471934, China
[3] School of Chemistry and Chemical Engineering, Luoyang Normal University, Luoyang 471934, China; lxm-nanoenergy@lynu.edu.cn

* Correspondence: zjg_1981@163.com (J.Z.); 11226019@zju.edu.cn (H.J.)

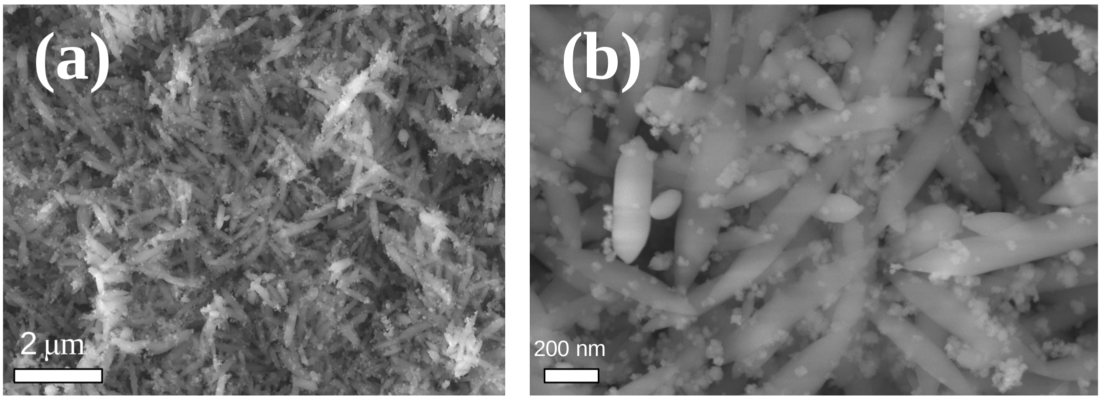

Figure S1. SEM images of (**a**) low- and (**b**) high-magnification of the Fe-MOFs nanorods.

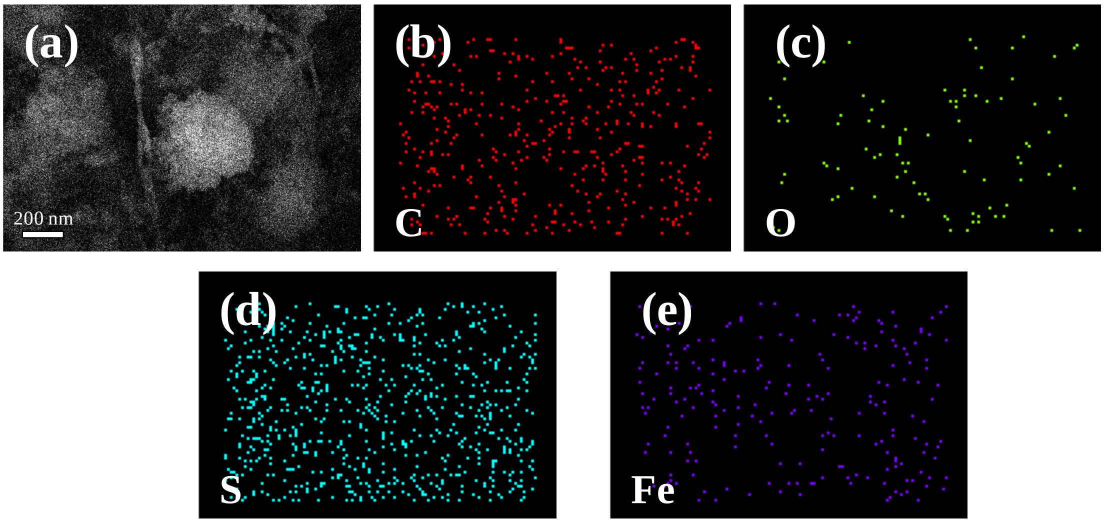

Figure S2. The elemental mapping images of FeS/C nanosheets.

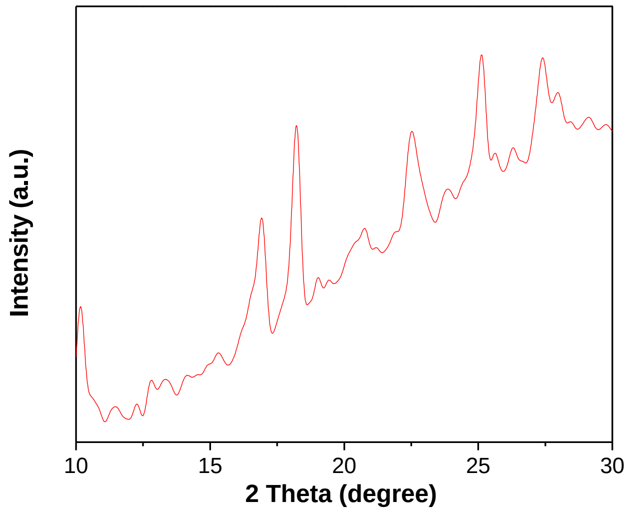

Figure S3. XRD pattern of uniform Fe-MOF nanorods.

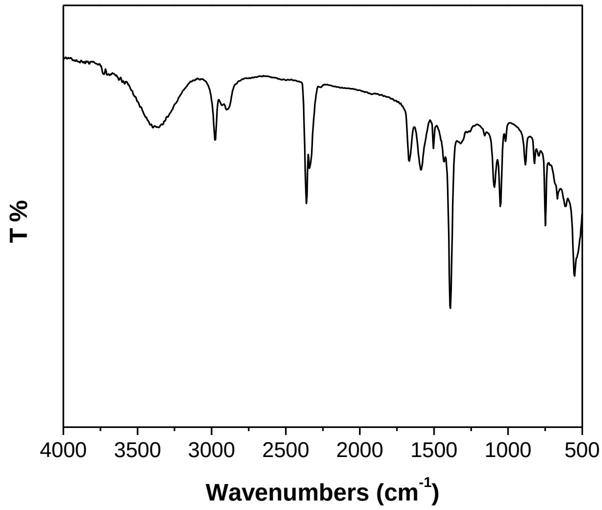

Figure S4. FTIR pattern of uniform Fe-MOF nanorods.

Table S1. Recent reports on the electrochemical data of FeS as anode material for lithium-ion batteries

Composite	Preparation method	Current density	Cycle number	Capacity (mAh/g)	References
FeS/porous carbon	One-pot solid-state method	0.1C	150	624.9	1
FeS@CNFs	Electrospinning	0.5A/g	200	535.1	2
FeS@RGO	Direct-precipitation	2C	300	283	3
FeS@C/carbon cloth	Hydrothermal	0.15C	100	420	4
FeS microsheet	Solution-based approach	0.1A/g	20	697	5
FS-ND ⊂ PGC-NW	Electrospinning	0.5 C	50	400	6
FeS@CNS	Freeze-drying/carbonization method	1 A/g	150	703	7
G@FeS-GNRs	Solution-based chemical unzipping	0.4A/g	100	536	8
FeS@RGO nanoparticles	Ionic liquid assisted solid-state method	0.1A/g	40	978	9
FeS/C nanosheets	Solid-state direct sulfuration	0.1A/g	150	830	This work

References:

1. S. P. Guo, J. C. Li, Z. Ma, Y. Chi, and H. G. Xue, A facile method to prepare FeS/porous carbon composite as advanced anode material for lithium-ion batteries, J. Mater. Sci. 54 (2017) 2345.

2. L. Fei, B. P. Williams, S. H. Yoo, J. M. Carlin, and Y. L. Joo, A general approach to fabricate free-standing metal sulfide@carbon nanofiber networks as lithium ion battery anodes. Chem. Commun. 52 (2016) 1501.

3. E. Shangguan, L. Guo, F. Li, Q. Wang, J. Li, Q. Li, Z. Chang, and X. Z. Yuan, FeS anchored reduced graphene oxide nanosheets as advanced anode material with superior high-rate performance for alkaline secondary batteries, J. Power Sources 327 (2016) 187.

4. X. Wei, W. Li, J. Shi, L. Gu, and Y. Yu, FeS@C on Carbon Cloth as Flexible Electrode for Both Lithium and Sodium Storage, ACS Appl. Mater. Interfaces 7 (2015) 27804.

5. C. Xing, D. Zhang, K. Cao, S. Zhao, X. Wang, H. Qin, J. Liu, Y. Jiang, and L. Meng, In Situ Growth of FeS Microsheet Networks with Enhanced Electrochemical Performance for Lithium-Ion Batteries, J. Mater. Chem. A 3 (2015) 8742.

6. C. Zhu, Y. Wen, P. A. van Aken, J. Maier, and Y. Yu, High Lithium Storage Performance of FeS Nanodots in Porous Graphitic Carbon Nanowires, Adv. Funct. Mater. 25 (2015) 2335.

7. Y. Xu, W. Li, F. Zhang, X. Zhang, W. Zhang, C. S. Lee, and Y. Tang, In situ incorporation of FeS nanoparticles/carbon nanosheets composite with an interconnected porous structure as a high-performance anode for lithium ion batteries, J. Mater. Chem. A 4 (2016) 3697.

8. L. Li, C. Gao, A. Kovalchuk, Z. Peng, G. Ruan, Y. Yang, H. Fei, Q. Zhong, Y. Li, and J. M. Tour, Sandwich structured graphene-wrapped FeS-graphene nanoribbons with improved cycling stability for lithium ion batteries, Nano Res. 9 (2016) 2904.

9. L. Fei, Q. L. Lin, B. Yuan, G. Chen, P. Xie, Y. L. Li, Y. Xu, S. D. Deng, S. Smirnov, and H. M. Luo, Reduced graphene oxide wrapped FeS nanocomposite for lithium-ion battery anode with improved performance. ACS Appl Mater 5 (2013) 5330.
